# Special Issue “Molecular Basis of Inherited Diseases in Companion Animals”

**DOI:** 10.3390/genes12010068

**Published:** 2021-01-07

**Authors:** Steven G. Friedenberg, Danika L. Bannasch

**Affiliations:** 1 Department of Veterinary Clinical Sciences, College of Veterinary Medicine, University of Minnesota, Saint Paul, MN 55108, USA; 2 Department of Population Health & Reproduction, School of Veterinary Medicine, University of California Davis, Davis, CA 95616, USA; dlbannasch@ucdavis.edu

The study of inherited diseases in companion animals has exploded over the past 15 years since the publication of the first dog genome in 2005 [1] and the cat genome in 2007 [2]. Since then, countless tools and resources have been developed allowing researchers to exploit these genomes to study inherited diseases and traits in companion animals at an unprecedented pace. According to the Online Mendelian Inheritance in Animals (OMIA) database [3], as of December 2020, there are 784 single-locus diseases or traits that have been explained in dogs and 361 in cats. Identification of the genetic polymorphisms that underlie these diseases and traits has allowed us to reduce the incidence of many inherited disorders and explain much of the phenotypic diversity seen in our companion animals. Furthermore, many of these now well-characterized inherited diseases in companion animals offer potential models for similar conditions in humans.

One notable trend in companion animal genetics over the past several years has been the rapidly increasing use of whole-genome resequencing as a tool for identifying genetic variants associated with disease. Indeed, the manuscripts that comprise this Special Issue reflect this ongoing trend: Of the 15 articles in this issue, 11 employed whole-genome resequencing to identify likely causative mutations. What is even more remarkable, however, is that over half of these 11 manuscripts employed whole-genome resequencing exclusively as a means of identifying putative causative mutations without making use of traditional marker arrays.

A major driver of this trend is undoubtedly the rapidly falling costs of whole-genome resequencing, along with the increasing availability of computational resources required to process and analyze these large datasets. Perhaps an even more important driver, however, has been the development of consortium-driven resources to pool and share whole-genome resequencing data with investigators around the world. This includes resources such as the 99 Lives Cat Genome Consortium [4] and the Dog Variant Database and Biomedical Consortium [5]. These databases of known genetic variation allow researchers to quickly compare a particular genome of interest to hundreds or thousands of already sequenced animals in order to determine whether a potentially pathogenic allele is unique to an animal with a particular trait or condition. This process allows for a rapid filtering of millions of variants to hundreds or tens of variants that can then be prioritized rapidly based upon the currently understood function of a particular gene. Next-generation reference genomes built using long-range sequencing technology [6,7], along with ever-improving genome annotations, are also rapidly improving the feasibility of using whole-genome resequencing to identify variants of interest for a particular trait or condition.

A natural consequence of the increasing use of whole-genome resequencing for our companion animals is the opportunity to provide truly precision medicine for individual patients. As veterinarians and geneticists, we are often confronted with unique cases with abnormalities that may be specific to a particular animal. In some cases, sequencing the animal’s entire genome may provide an opportunity to arrive at a diagnosis in a way that traditional medical testing could not. This trend toward precision medicine is also reflected in this Special Issue, as four articles identified disease-causing mutations that are believed to be specific to only one animal. As whole-genome resequencing costs continue to decline and this technique is even more widely applied in veterinary hospitals around the world, we will begin to gain a better grasp on understanding the breadth of disorders we see among companion animals, along with their underlying genetic roots. Undoubtedly, this work will also lead to new spontaneous models of animal disease that can inform our understanding of similar diseases in humans and other species as well.

Despite the advances afforded by whole-genome resequencing, one area of genetics where this technology alone is unlikely to provide a complete understanding is complex inherited traits. These traits, which are polygenic by nature, are likely to continue to require a combination of marker arrays, whole-genome resequencing, and other approaches such as selection mapping in order to fully understand the contribution of genetic variation to the incidence of disease. Two of the articles in this Special Issue, one on diabetes [8] and the other on obesity [9], reflect the challenges associated with understanding the genetic basis of complex traits in companion animals. Because many common diseases we see in companion animal medicine are likely complex traits (e.g., autoimmune disorders, breed-associated cancers), these areas remain ripe for ongoing research as we continue to improve the tools and resources we have at our disposal as geneticists.

One last point regarding the articles in this Special Issue which we would be remiss to overlook is the degree of overlap between companion animal and human disorders. In fact, all 15 manuscripts in this Special Issue describe inherited disorders in companion animals with parallels in humans. This observation underscores the value of ongoing “One Health” approaches to medicine, which are meant to recognize the connections between the health of people, animals, and the environment. Notably, in this Special Issue, these connections were made by collaborations between veterinarian scientists and, in most cases, Ph.D. scientists. That all of these manuscripts were co-authored by veterinarians highlights the many advantages of dogs and cats over more traditional model organisms: a shared living environment, the breadth and depth of quality medical diagnostics and treatments, and the dedication of animal owners around the world that continues to drive this field forward. The disease parallels between animals and humans highlight the importance of companion animals in providing sources of spontaneous disease models for similar conditions in humans that would be difficult to re-create in a laboratory setting.

In summary, this issue celebrates the rapidly growing and evolving field of companion animal genetics by demonstrating how cutting-edge tools can be employed to help us understand the inherited basis of diseases. As new reference genomes and improved sequencing technologies continue to emerge and enhance our ability to understand inherited disorders, we are confident that many more exciting discoveries in the world of companion animal genetics are certain to emerge.

## References

[1] Lindblad-Toh K., Wade C.M., Mikkelsen T.S., Karlsson E.K., Jaffe D.B., Kamal M., Clamp M., Chang J.L., Kulbokas E.J., Zody M.C. (2005). Genome sequence, comparative analysis and haplotype structure of the domestic dog. Nature.

[2] Pontius J.U., Mullikin J.C., Smith D.R., Team A.S., Lindblad-Toh K., Gnerre S., Clamp M., Chang J., Stephens R., Neelam B. (2007). Initial sequence and comparative analysis of the cat genome. Genome Res..

[3] OMIA—Online Mendelian Inheritance in Animals. https://www.omia.org/home/.

[4] Lyons Feline & Comparative Genetics—Lyons’ Den at the University of Missouri. https://felinegenetics.missouri.edu/.

[5] Jagannathan V., Drögemüller C., Leeb T. (2019). Dog Biomedical Variant Database Consortium (DBVDC). A comprehensive biomedical variant catalogue based on whole genome sequences of 582 dogs and eight wolves. Anim. Genet..

[6] Buckley R.M., Davis B.W., Brashear W.A., Farias F.H.G., Kuroki K., Graves T., Hillier L.W., Kremitzki M., Li G., Middleton R. (2020). A new domestic cat genome assembly based on long sequence reads empowers feline genomic medicine and identifies a novel gene for dwarfism. PLoS Genet..

[7] Wang C., Wallerman O., Arendt M.-L., Sundström E., Karlsson Å., Nordin J., Mäkeläinen S., Pielberg G.R., Hanson J., Ohlsson Å. (2020). A new long-read dog assembly uncovers thousands of exons and functional elements missing in the previous reference. bioRxiv.

[8] Balmer L., O’Leary C.A., Menotti-Raymond M., David V., O’Brien S., Penglis B., Hendrickson S., Reeves-Johnson M., Gottlieb S., Fleeman L. (2020). Mapping of Diabetes Susceptibility Loci in a Domestic Cat Breed with an Unusually High Incidence of Diabetes Mellitus. Genes.

[9] Wallis N., Raffan E. (2020). The Genetic Basis of Obesity and Related Metabolic Diseases in Humans and Companion Animals. https://www.preprints.org/manuscript/202010.0301/v1.

